# Mental Health Status of Teachers During the Second Wave of the COVID-19 Pandemic: A Web-Based Study in Bangladesh

**DOI:** 10.3389/fpsyt.2022.938230

**Published:** 2022-07-25

**Authors:** Md. Tanvir Hossain, Md. Akhtarul Islam, Nusrat Jahan, Mst. Tanmin Nahar, Md. Juwel Ahmed Sarker, Md. Mostafizur Rahman, Farah Deeba, Kazi Enamul Hoque, Rina Aktar, Md. Mazharul Islam, Mohammed Zaber Hossain, Laila Siddiqua, Zisan Mahbub, Md. Nazrul Islam

**Affiliations:** ^1^Sociology Discipline, Social Science School, Khulna University, Khulna, Bangladesh; ^2^Statistics Discipline, Science, Engineering and Technology School, Khulna University, Khulna, Bangladesh; ^3^Department of Development Studies, Faculty of Social Science and Humanities, Hajee Mohammad Danesh Science and Technology University, Dinajpur, Bangladesh; ^4^Department of Disaster and Human Security Management, Faculty of Arts and Social Sciences, Bangladesh University of Professionals, Dhaka, Bangladesh; ^5^Department of Clinical Psychology, Faculty of Biological Sciences, University of Dhaka, Dhaka, Bangladesh; ^6^Faculty of Education, University of Malaya, Kuala Lumpur, Malaysia; ^7^Department of Sociology, Faculty of Social Science, Government Azizul Haque College, Bogura, Bangladesh; ^8^Department of English, Bangladesh Military Academy, Bhatiari, Bangladesh; ^9^Architecture Discipline, Science, Engineering and Technology School, Khulna University, Khulna, Bangladesh; ^10^Upazila Health Complex, Dumuria, Bangladesh; ^11^Forestry and Wood Technology Discipline, Life Science School, Khulna University, Khulna, Bangladesh

**Keywords:** COVID-19, Bangladesh, teacher, depression, anxiety, stress, prevalence, mental health

## Abstract

**Background:**

Following the outbreak of the COVID-19 pandemic, the government of Bangladesh implemented strict non-therapeutic measures, i.e., “social distancing,” “lockdown,” “work from home,” in the first quarter of 2020. Like other professionals, teachers at schools, colleges and universities were confined within households. However, the introduction of online education imposed an additional burden on teachers along with growing household responsibilities, thus, affecting their psychological state.

**Aims:**

This study was aimed to explore the prevalence of mental health problems among teachers in Bangladesh and to identify the associated risk factors.

**Methods:**

This web-based cross-sectional study was conducted during the second wave of COVID-19 pandemic in Bangladesh. Data were collected from 381 teachers working at schools, colleges, and universities between 01 August and 29 August 2021 by administering a self-reported e-questionnaire using Google Form, where the mental health of teachers was assessed by depression, anxiety, and stress scale. Data were analyzed using IBM SPSS Statistics (Version 26) and STATA Version 16, and multiple linear regression was executed to predict mental health problems among teachers.

**Results:**

The findings indicate that the overall prevalence of depression, anxiety, and stress among teachers was 35.4%, 43.7%, and 6.6%, respectively. The prevalence was higher among male and older teachers than among their female and younger colleagues. The findings further showed that place of residence, institution, self-reported health, usage of social and electronic media, and fear of COVID-19 significantly influenced the mental health status of teachers.

**Conclusion:**

It is strongly recommended that the government and policymakers provide proper mental health services to teachers in order to reduce mental health problems and thus sustain the quality of education during and after the pandemic.

## Introduction

In late December 2019, the coronavirus disease (COVID-19) spread across China from Wuhan (1). Toward the end of January 2020, the World Health Organization (WHO) issued a warning about a public health emergency (2); and they declared a pandemic on 11 March 2020 (3). As of June 2022, around 6.3 million people have died of COVID-19, and it has infected over 533 million people worldwide (4). Healthcare systems were overwhelmed with infected and suspected COVID-19 patients in both developed and developing countries, leading to disarray in social, economic, educational, and political systems (5–7) and burdening the mental health of individuals, families, communities, societies, and countries across the world (8, 9).

Although government and international organizations immediately implemented non-therapeutic preventive and protective measures, including “social distancing,” “lockdown” or “home confinement,” and “face mask” (10, 11), the news of growing infections and death across the world caused panic among people (12). The situation was further worsened by exposure to “misinformation” on social and electronic media such as Facebook, Messenger, WhatsApp, Twitter, and television (13, 14). Thus, many people, irrespective of age, sex, occupation or region, experienced heightened mental health problems, including depression, anxiety, stress, fear, and poor sleep (15, 16); some even committed suicide (17, 18).

In Bangladesh, out of 1.9 million confirmed cases, over 29,000 people have died as of June 2022 (19). Since 2020, numerous studies have been carried out in Bangladesh to assess the impact of COVID-19 on the mental health of different occupational groups and cohorts, including medical professionals (20–22), marginalized workers (5, 23), children (24, 25), college and university students (26–28), and middle-aged and older adults (29). For example, a study on students in May 2020 revealed that the prevalence of anxiety and depression among university students was 81.7% and 82.4%, respectively, (28), while another study suggested that 27.1% of university students experienced poor sleep quality during April 2020 (27). Likewise, doctors also experienced heightened depression (55.3%), anxiety (48.4%), and stress (35.2%) during the pandemic (20).

Although a good number of studies focusing on the mental health condition of teachers have been conducted in other parts of the world (30–35), to the best of our knowledge, there has been none in Bangladesh. Teachers worldwide have been continuously working online under unfavorable circumstances during the pandemic in order to minimize the mental health burden of students and their guardians (36, 37). In doing so, teachers have experienced an intensified psychological problem. For example, a study in India found that growing household responsibilities, e.g., home management, childcare, and elderly care, followed by the introduction of online platform-based education increased work burden and stress among female teachers; this adversely affected their psychological state, eventually leading to irritation and aggressive behavior (30). Studies in China reported growing anxiety and post-traumatic stress disorder (PTSD) among teachers in schools, colleges, and universities (34, 35).

Studies in western Europe have also identified volatile mental health conditions among teachers (31–33, 38, 39). Studies in Spain reported heightened depression, anxiety, and stress, particularly among female and older teachers and among teachers suffering from chronic diseases or living with chronically ill or COVID-19 infected family members (33, 38). Furthermore, work stability also affected the magnitude of mental health problems among teachers (38). Meanwhile, studies in England found that growing uncertainty, together with an increased workload, health vulnerabilities, exposure to non-stop negative news in media, and concern over the wellbeing of students and colleagues, negatively affected the mental health and wellbeing of primary and secondary teachers (31, 32). A Polish study on primary and secondary teachers showed that mental health problems rose to over 50% during the second phase of the COVID-19 pandemic among teachers for depression (54.99%), anxiety (50.73%), and stress (55%), respectively; number of children, partner employment status, and changes in quality and satisfaction of relationship were key determinants of depression, anxiety, and stress symptoms among the Polish teachers (39). Similarly, studies in the North and South America suggested that unpaid work overload, sense of uncertainty, home confinement-induced loneliness, loss of loved ones, and fear of the pandemic significantly deteriorated the mental health conditions of teachers, particularly female and older teachers (40–42). The lower mental health status of teachers subsequently affected their self-rated health status (43), leading to poor sleep and appetite, increased headaches and stomachaches and drug use, and distrust among teachers that compelled them to think about leaving the teaching profession (43, 44).

The aforementioned studies clearly suggest that teachers across the world have been experiencing increased mental health problems, including depression, anxiety, and stress; this is caused by a wide variety of factors, such as personal characteristics (age, sex, marital status, and race), socioeconomic status (work experience and levels, family composition) work, and COVID-19 related health issues (COVID-19 infection, loss of family members and friends, exhaustion, and health issues) which significantly determine the presence and absence of mental health problems. However, there has been a dearth of empirical studies in the context of Bangladesh; as a result, the present study aimed to assess the prevalence of mental health problems among teachers in Bangladesh and to identify the associated risk factors. This study may guide the government and its policymakers to formulate new policies and strategies for safeguarding the mental wellbeing of teachers in Bangladesh and in other parts of the world.

## Materials and Methods

### Study Settings and Participants

**MAP 1:**
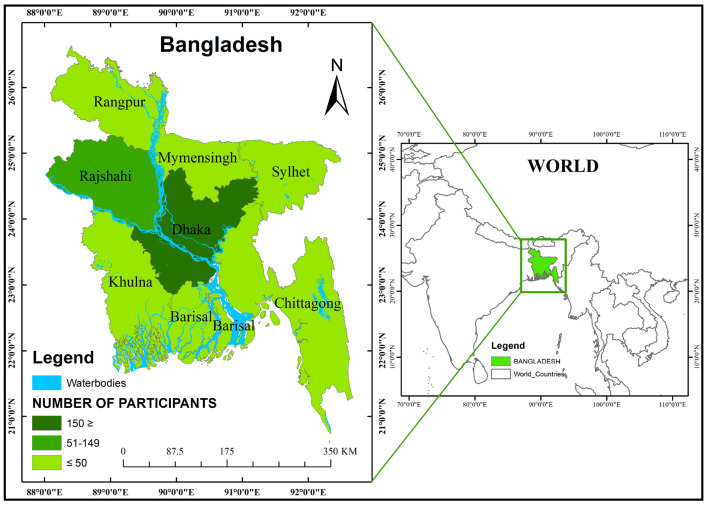
Study area with distribution of participants by division.

This cross-sectional web-based survey was adopted to assess the prevalence of depression, anxiety, and stress among teachers during the second wave of the COVID-19 pandemic when complying with the “social distancing” recommended by the World Health Organization. Data were collected from teachers in various schools, colleges, and universities, using an anonymous semi-structured electronic questionnaire (e-questionnaire) in English through Google Form. The inclusion criteria of the participants were – (i) Bangladeshi citizens, (ii) adults (≥18 years of age), (iii) living in Bangladesh during the second wave of COVID-19; (iv) employed as permanent teachers in their respective educational institutions; and (v) holding an active/valid email or social media account (Facebook/Messenger/WhatsApp). Considering these specifications, the researchers collected the e-questionnaire responses through institutional email and social media groups, and each participant was requested to share the e-questionnaire with their colleagues and friends; thus, this study followed the convenience sampling technique. It is important to note that around one million teachers are involved in teaching and learning at all three levels in Bangladesh (45, 46), of which a lion’s share do not have access to the internet or email or social media account (47). In this study, however, a total of 439 responses were initially recorded between 01 August and 29 August 2021. Fifty-eight responses were excluded due to incompleteness or repetitive responses; therefore, responses from 381 teachers were eventually included in this study (see Map 1). In order to ensure transparency and to eliminate repeated question items, the e-questionnaire was pre-tested on a small group of teachers (20), working at different schools, colleges, and universities. The responses from the participants of the pre-test were excluded from the web-based survey to avoid biases.

### Ethical Issues

This study was approved by the Ethical Clearance Committee of Khulna University, Bangladesh (Reference No. KUECC – 2021/09/26). In the first section of the e-questionnaire, an informed consent letter was attached where the participants responded anonymously by filling out the form. In the consent form, all participants were provided with information concerning the research purpose, confidentiality, and the right to revoke participation without prior justification. There was no incentive for the participants.

### Measures

#### Socio-Demographic Information

The initial section of the e-questionnaire contained the socio-demographic information of the teachers, including their current age (“≤30,” “31 – 40,” or “≥41”), sex (“female” or “male”), place of residence (“rural” or “urban”), marital status (“unmarried,” “married,” or “others [widowed/widower or divorced/separated]”), type of institution at which they were working (“school,” “college,” or “university”) higher educational degree (“Master” or “PhD”), designation (“assistant/senior teacher/demonstrator,” “lecturer/assistant professor,” or “associate professor/professor”), self-reported health (“poor/fair,” “good,” “very good,” or “excellent”), COVID-19 testing status to find out whether s/he was infected or not (“not tested” or “tested”), vaccine status (“no” or “yes”), fear of COVID-19 (“not fearful” or “fearful”), exposure to information regarding COVID-19 (“never/sometimes/occasionally” or “often/always”), and usage of social and electronic media (“decreased,” “about the same,” or “increased”).

#### Depression, Anxiety, Stress Scale 21

The mental health of teachers was assessed by a widely used scale – depression, anxiety, stress scale (DASS) – developed in the mid-90s (48). The initial DASS comprised 42 items in a four-point Likert scale to measure the negative emotional states of depression, anxiety, and stress, 14 questions for each sub-scale (48), and later a short version of the DASS – the depression, anxiety, and stress scale 21 (DASS 21) – was developed and validated (49), where each sub-scale of depression, anxiety and stress consisted of seven items. The DASS 21 in English was used in this study considering the educational qualifications of teachers. A sum of scores of the seven items in each sub-scale was estimated to measure the presence and absence of depression, anxiety, and stress. The sum of scores ≥ 10 indicates the presence of depression, whereas it was ≥8 and ≥15 for anxiety and stress, respectively. The overall Cronbach’s alpha (α) of the DASS 21 was 0.950, an excellent internal consistency (50), while the Cronbach’s α of each sub-scale was α = 0.885 (depression), α = 0.847 (anxiety) and α = 0.877 (stress), respectively.

### Analysis

Data were analyzed in two consecutive phases using IBM SPSS Statistics (Version 26) and STATA version 16 for windows. Descriptive statistics, i.e., frequency and percentage analysis, were calculated to present the socio-demographic information of the participants. The prevalence of depression, anxiety, and stress was estimated with standard error (SE). The simple linear regression (SLR) and multiple linear regression (MLR) analysis with unstandardized (B) and standardized Coefficient (β), at 95% confidence interval (CI; 51, 52) were utilized to identify the risk factors associated with mental health problems of teachers, e.g., depression, anxiety, and stress. Different factors were considered to be statistically significant when the *p* value was < 0.05.

## Results

### Descriptive Information of the Participants

Table 1 demonstrates the socio-demographic information of the participating teachers. Among the participants, more than 80% were older than 30 years and married, while more than half were male and lived in urban areas. Around 94% had a master’s degree, however, working mostly in colleges and schools. Less than 10% of the participants reported having an “excellent” health status, and less than a quarter assess their COVID-19 infection status during the pandemic. Around 88% of teachers were vaccinated, and 62.2% of them admitted increasing social and electronic media usage during the pandemic.

**TABLE 1:** Descriptive information of the participants (*n* = 381).

Variable	Frequency (%)
**Age**	
≤30	51 (13.4%)
31–40	163 (42.8%)
≥41	167 (43.8%)
**Sex**	
Female	149 (39.1%)
Male	232 (60.9%)
**Place of residence**	
Rural	158 (41.5%)
Urban	223 (58.5%)
**Marital status**	
Unmarried	35 (9.2%)
Married	336 (88.2%)
Others	10 (2.6%)
**Institution**	
School	129 (33.9%)
College	143 (37.5%)
University	109 (28.6%)
**Education**	
Master	357 (93.7%)
PhD	24 (6.3%)
**Designation**	
Assistant/senior teacher/demonstrator	128 (33.6%)
Lecturer/Assistant Professor	176 (46.2%)
Associate Professor/Professor	77 (20.2%)
S**elf-reported health**	
Poor/Fair	41 (10.8%)
Good	201 (52.8%)
Very good	109 (28.6%)
Excellent	30 (7.9%)
**COVID-19 testing status**	
Not tested	297 (78.0%)
Tested	84 (22.0%)
**Vaccine status**	
No	46 (12.1%)
Yes	335 (87.9%)
**Fear of COVID-19**	
Not fearful	174 (45.7%)
Fearful	207 (54.3%)
**Exposure to information**	
Never/sometimes/occasionally	150 (39.4%)
Often/always	231 (60.6%)
**Usage of social and electronic media**	
Decreased	86 (22.6%)
About the same	58 (15.2%)
Increased	237 (62.2%)

### Prevalence of Depression, Anxiety, and Stress Among Teachers During the COVID-19 Pandemic

**FIGURE 1:**
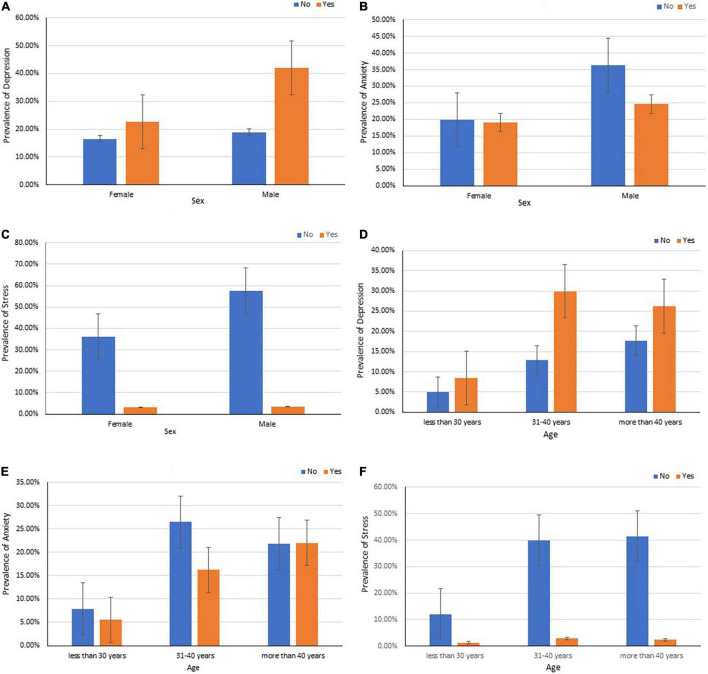
Prevalence and standard error of depression **(A,D)**, anxiety **(B,E)**, and stress **(C,F)** among teachers in Bangladesh by sex and age.

The overall prevalence of depression, anxiety, and stress among teachers in Bangladesh was 35.4% (SE 0.024), 43.7% (SE 0.025), and 6.6% (SE 0.012), respectively. The prevalence was higher for male teachers in depression (42% vs. 22.6%), anxiety (24.6% vs. 19.1%), and stress (3.4% vs. 3.1%) than female teachers (see Figure 1). It is also apparent from Figure 1 that depression (29.9%) and stress (2.9%) were higher among teachers with age between 31 to 40 years, while teachers older than 40 years of age showed greater anxiety symptoms during the COVID-19 pandemic in Bangladesh.

### Determinants of Depression Among Teachers During the COVID-19 Pandemic

Table 2 indicates the SLR and MLR models with unstandardized and standardized coefficients with 95% CI. In the MLR model, place of residence, institution, self-reported health, fear of COVID-19, and usage of social and electronic media were found to be significantly associated with depression among teachers during the COVID-19 pandemic [*R*^2^ Adjusted = 0.378, *F* (20,360) = 10.95, *p* < 0.000]. Depression among the teachers from the urban area was 1.37 units lower than the teachers from rural areas. Teachers who reported having good and excellent health were 2.06 units and 2.35 units less likely to be depressed, respectively, as opposed to teachers with poor/fair health status. College teachers showed less depression compared to schoolteachers. Teachers who feared COVID-19 revealed a 2.4 unit increase in depression compared to those who did not fear the COVID-19; on the other hand, there exists a 2.73 units reduction in depression for the teacher who has increased the use of social and electronic media. Moreover, participants who maintained a stable social and electronic media usage showed a reduction in depressive symptoms.

**TABLE 2:** Predicting depression among teachers during the COVID-19 pandemic.

Variable	Simple linear regression model						Multiple linear regression model					
		
	B	β	*t*	*P*-value	95% CI		B	β	*t*	*P*-value	95% CI	
										
					Lower value	Upper value					Lower value	Upper value
**Age**												
≤30 ^ref^												
31–40	–1.840	–0.181	–2.29	0.023	–3.420	–0.260	–1.509	–0.148	–1.88	0.060	–3.085	0.066
≥41	–0.796	–0.079	–0.99	0.321	–2.371	0.780	–1.435	–0.142	–1.60	0.110	–3.199	0.327
**Sex**												
**Female ^ref^**												
Male	–1.514	–0.147	–2.89	0.004	–2.544	–0.484	–0.805	–0.078	–1.77	0.077	–1.69	0.087
**Place of residence**												
**Rural^ref^**												
Urban	–3.747	–0.367	–7.68	0.000	–4.707	–2.788	–1.370	–0.134	–2.39	0.017	–2.497	–0.243
**Marital status**												
**Unmarried ^ref^**												
Married	–0.730	–0.047	–0.81	0.416	–2.491	1.033	–0.406	–0.026	–0.51	0.611	–1.977	1.164
Others	0.214	0.007	0.12	0.906	–3.343	3.771	0.678	0.022	0.45	0.656	–2.316	3.672
**Institution**												
**School ^ref^**												
College	–5.498	–0.529	–10.14	0.000	–6.563	–4.432	–2.530	–0.243	–2.55	0.011	–4.483	–0.576
University	–3.798	–0.341	–6.54	0.000	–4.940	–2.656	–1.620	–0.146	–1.51	0.133	–3.736	0.495
**Education**												
**Master ^ref^**												
PhD	–3.182	–0.153	–3.03	0.003	–5.249	–1.115	–1.259	–0.061	–1.01	0.315	–3.718	1.199
**Designation**												
**Assistant/senior teacher/demonstrator ^ref^**												
Lecturer/Assistant Professor	–2.876	–0.285	–5.58	0.000	–3.889	–1.863	0.516	0.051	0.70	0.487	–0.943	1.975
Associate Professor/Professor	–4.285	–0.246	–4.82	0.000	–6.033	–2.536	0.657	0.038	0.52	0.602	–1.823	3.139
**Self-reported health**												
**Poor/Fair ^ref^**												
Good	–0.339	–0.034	–0.39	0.694	–2.036	1.356	–1.179	–0.117	-1.57	0.118	–2.661	0.302
Very good	–1.244	–0.112	–1.35	0.178	–3.058	0.570	–2.063	-0.185	-2.54	0.011	-3.658	–0.467
Excellent	0.1626	0.008	0.13	0.893	–2.216	2.541	–2.352	–0.126	–2.23	0.026	–4.426	–0.279
**COVID-19 testing status**												
**Not tested ^ref^**												
Tested	–2.511	–0.207	–4.12	0.000	–3.712	–1.312	–0.978	–0.081	–1.82	0.069	–2.035	0.077
**Vaccine status**												
**No ^ref^**												
Yes	0.416	0.027	0.52	0.600	–1.143	1.975	0.090	0.006	0.12	0.902	–1.349	1.530
**Fear of COVID-19**												
**Not fearful ^ref^**												
Fearful	4.112	0.407	8.68	0.000	3.181	5.045	2.402	0.238	5.08	0.000	1.472	3.333
**Exposure to information**												
**Never/sometimes/occasionally ^ref^**												
Often/always	2.202	0.214	4.26	0.000	1.185	3.218	0.150	0.015	0.32	0.749	–0.773	1.074
**Usage of social and electronic media**												
**Decreased ^ref^**												
About the same	–4.753	–0.339	–6.07	0.000	–6.291	–3.214	–2.130	–0.152	–2.68	0.008	–3.696	–0.565
Increased	–4.991	–0.481	–8.61	0.000	–6.131	–3.852	–2.727	–0.262	–4.03	0.000	–4.059	–1.394

*^B^Unstandardized Coefficient, ^β^Standardized Coefficient, ^CI^Confidence interval, and ^ref^Reference category.*

### Determinants of Anxiety Among Teachers During the COVID-19 Pandemic

Table 3 demonstrated that the MLR model is significant [*R*^2^ Adjusted = 0.297, *F* (20,360) = 9.01, *p* < 0.000] and indicated a significant association of teachers’ institution, fear of COVID-19, and social and electronic media usage with anxiety among teachers during the COVID-19 pandemic. College teachers and university teachers showed a 2.76 and 2.45 unit decrease in anxiety symptoms, respectively, compared that to the schoolteachers. Teachers with a heightened fear of COVID-19 experienced a 2.18 unit increase in anxiety level compared to those not afraid of COVID-19. Teachers experiencing an increase in social and electronic media usage had a 1.73 units reduction in anxiety compared to teachers who reduced the use of social and electronic media during this pandemic.

**TABLE 3:** Predicting anxiety among teachers during the COVID-19 pandemic.

Variable	Simple linear regression model						Multiple linear regression model					
		
	B	β	*t*	*P*-value	95% CI		B	β	*t*	*P*-value	95% CI	
										
					Lower value	Upper value					Lower value	Upper value
**Age**												
**≤30 ^ref^**												
31–40	–0.972	–0.102	–1.29	0.199	–2.459	0.514	–1.467	–0.154	–1.88	0.060	–2.998	0.064
≥41	0.068	0.007	0.09	0.928	–1.414	1.551	–1.362	–0.143	–1.56	0.119	–3.076	0.351
**Sex**												
**Female ^ref^**												
Male	–0.877	–0.091	–1.77	0.077	–1.850	0.096	–0.291	–0.030	–0.66	0.509	–1.158	0.576
**Place of residence**												
**Rural^ref^**												
Urban	–3.403	–0.355	–7.39	0.000	–4.308	–2.498	–0.916	–0.096	–1.65	0.101	–2.011	0.178
**Marital status**												
**Unmarried ^ref^**												
Married	–0.097	–0.007	–0.12	0.908	–1.753	1.558	–0.130	–0.009	–0.17	0.867	–1.656	1.396
Others	0.457	0.015	0.27	0.788	–2.885	3.799	0.774	0.026	0.52	0.601	–2.135	3.684
**Institution**												
**School ^ref^**												
College	–5.127	–0.526	–10.16	0.000	–6.119	–4.135	–2.759	–0.282	–2.86	0.005	–4.657	–0.861
University	–4.272	–0.409	–7.91	0.000	–5.335	–3.209	–2.447	–0.234	–2.34	0.020	–4.503	–0.391
**Education**												
**Master ^ref^**												
PhD	–3.372	–0.173	–3.43	0.001	–5.306	–1.439	–1.889	–0.097	–1.55	0.121	–4.278	0.500
**Designation**												
**Assistant/senior teacher/demonstrator ^ref^**												
Lecturer/Assistant Professor	–3.149	–0.332	–6.62	0.000	–4.085	–2.214	0.235	0.025	0.33	0.745	–1.182	1.653
Associate Professor/Professor	–4.352	–0.266	–5.30	0.000	–5.967	–2.737	0.809	0.049	0.66	0.510	1.602	3.220
**Self-reported health**												
**Poor/Fair ^ref^**												
Good	0.750	0.079	0.92	0.356	–0.845	2.346	–0.232	–0.025	–0.32	0.751	–1.672	1.207
Very good	0.505	0.048	0.58	0.561	–1.201	2.212	–0.563	–0.054	–0.72	0.475	–2.113	0.986
Excellent	1.380	0.079	1.21	0.226	–0.857	3.618	–0.952	–0.054	–0.93	0.353	–2.966	1.062
**COVID-19 testing status**												
**Not tested ^ref^**												
Tested	–2.366	–0.208	–4.13	0.000	–3.492	–1.241	–0.852	–0.075	–1.63	0.103	–1.878	0.173
**Vaccine status**												
**No ^ref^**												
Yes	1.037	0.072	1.40	0.163	–0.422	2.498	0.286	0.019	0.40	0.688	–1.112	1.685
**Fear of COVID-19**												
**Not fearful ^ref^**												
Fearful	3.704	0.391	8.26	0.000	2.822	4.585	2.183	0.230	4.75	0.000	1.279	3.087
**Exposure to information**												
**Never/sometimes/occasionally ^ref^**												
Often/always	1.905	0.197	3.91	0.000	0.948	2.863	0.091	0.009	0.20	0.840	–0.805	0.989
**Usage of social and electronic media**												
**Decreased ^ref^**												
About the same	–3.622	–0.276	–4.87	0.000	–5.086	–2.159	–0.721	–0.055	–0.93	0.352	–2.242	0.800
Increased	–4.427	–0.454	–8.03	0.000	–5.512	–3.343	–1.730	–0.177	–2.63	0.009	–3.025	–0.435

*^B^Unstandardized Coefficient, ^β^Standardized Coefficient, ^CI^Confidence interval, and ^ref^Reference category.*

### Determinants of Stress Among Teachers During the COVID-19 Pandemic

Table 4 indicated that the MLR model for stress is significant [*R*^2^ Adjusted = 0.312, *F* (20,360) = 9.63, *p* < 0.000] and showed that teachers’ age, institution, self-reported health status, fear of COVID-19, and usage of social and electronic media were significantly associated with stress among teachers during the COVID-19 pandemic. Findings demonstrated that teachers with the age group 31–40 years and 41 or more years of age were less stressed compared to teachers younger than 30 years of age. College teachers showed 2.23 units lower stress symptoms than schoolteachers during the COVID-19 pandemic. Compared to the teacher who did not have a COVID test, a teacher with a COVID test was 2.17 times less stressed. Teachers with good and excellent health status were 1.87 and 2.88 units less stressed, respectively. However, teachers with heightened fear of COVID-19 experienced a 2.37 unit increase in stress level compared to teachers who were not afraid of COVID-19. Findings also showed that teachers experienced 3.49 units and 2.95 units reduction in stress with increased usage of social and electronic media and keeping the same usage, respectively.

**TABLE 4:** Predicting stress among teachers during the COVID-19 pandemic.

Variable	Simple linear regression model						Multiple linear regression model					
		
	B	β	*t*	*P*-value	95% CI		B	β	*t*	*P*-value	95% CI	
										
					Lower value	Upper value					Lower value	Upper value
**Age**												
**≤30 ^ref^**												
31–40	–1.592	–0.159	–2.02	0.044	–3.141	–0.043	–1.708	–0.172	–2.13	0.034	–3.287	–0.130
≥41	–0.706	–0.071	–0.90	0.369	–2.250	0.838	–2.235	–0.225	–2.49	0.013	–4.002	–0.468
**Sex**												
**Female ^ref^**												
Male	–1.345	–0.133	–2.62	0.009	–2.354	–0.335	–0.602	–0.059	–1.32	0.186	–1.496	0.291
**Place of residence**												
**Rural^ref^**												
Urban	–2.960	–0.296	–6.04	0.000	–3.924	–1.996	–0.778	–0.078	–1.36	0.176	–1.907	0.349
**Marital status**												
**Unmarried ^ref^**												
Married	–0.211	–0.014	–0.24	0.810	–1.935	1.513	0.377	0.025	0.47	0.637	–1.195	1.950
Others	1.285	0.042	0.73	0.468	–2.195	4.766	1.868	0.061	1.22	0.221	–1.131	4.867
**Institution**												
**School ^ref^**												
College	–4.576	–0.450	–8.32	0.000	–5.657	–3.495	–2.229	–0.219	–2.24	0.026	–4.185	–0.272
University	–3.324	–0.305	–5.64	0.000	–4.482	–2.166	–1.897	–0.174	–1.76	0.079	–4.016	0.222
**Education**												
**Master ^ref^**												
PhD	–2.877	–0.142	–2.79	0.006	–4.903	–0.851	–1.880	–0.093	–1.50	0.134	–4.343	0.582
**Designation**												
**Assistant/senior teacher/demonstrator ^ref^**												
Lecturer/Assistant Professor	–2.188	–0.221	–4.24	0.000	–3.201	–1.173	1.322	0.134	1.78	0.076	–0.138	2.784
Associate Professor/Professor	–3.116	–0.183	–3.50	0.000	–4.866	–1.365	2.361	0.139	1.87	0.063	–0.124	4.846
**Self-reported health**												
**Poor/Fair ^ref^**												
Good	–0.153	–0.015	–0.18	0.857	–1.811	1.506	–0.761	–0.077	–1.01	0.314	–2.245	0.722
Very good	–1.242	–0.114	–1.38	0.169	–3.016	0.532	–1.874	–0.172	–2.31	0.022	–3.472	–0.276
Excellent	–0.395	–0.022	–0.33	0.738	–2.722	1.930	–2.877	–0.157	–2.72	0.007	–4.953	–0.800
**COVID-19 testing status**												
**Not tested ^ref^**												
Tested	–2.175	–0.183	–3.63	0.000	–3.354	–0.996	–0.862	–0.073	–1.60	0.110	–1.920	0.195
**Vaccine status**												
**No ^ref^**												
Yes	0.758	0.050	0.98	0.329	–0.766	2.283	0.768	0.051	1.05	0.295	–0.673	2.211
**Fear of COVID-19**												
**Not fearful ^ref^**												
Fearful	3.893	0.394	8.34	0.000	2.975	4.850	2.375	0.240	5.01	0.000	1.443	3.306
**Exposure to information**												
**Never/sometimes/occasionally ^ref^**												
Often/always	2.202	0.248	4.98	0.000	1.514	3.486	0.551	0.055	1.17	0.242	–0.373	1.477
**Usage of social and electronic media**												
**Decreased ^ref^**												
About the same	–4.857	–0.354	–6.35	0.000	–6.363	–3.352	–2.954	–0.216	–3.70	0.000	–4.522	–1.386
Increased	–4.946	–0.477	–8.55	0.000	–5.961	–3.731	–3.495	–0.344	–5.15	0.000	–4.830	–2.161

*^B^Unstandardized Coefficient, ^β^Standardized Coefficient, ^CI^Confidence interval, and ^ref^Reference category.*

## Discussion

This study aimed to assess the mental health status of teachers and to identify the possible risk factors. This study indicated that the prevalence of depression, anxiety, and stress among teachers was 35.4%, 43.7%, and 6.6%, respectively. The latest nationwide survey in 2019 suggested that the overall prevalence of depression, anxiety, and stress among the Bangladeshi population was 6.7%, 4.5%, and 2.1%, respectively, (53). It is, therefore, evident that teachers have experienced heightened mental health problems during the COVID-19 pandemic in Bangladesh. However, studies in Spain showed that the prevalence of mental health problems was more than 30% (depression) and 50% (anxiety and stress), respectively, among the Spanish teachers (33, 38). A two-phase study in Poland indicated that the mental health of teachers, e.g., depression, anxiety, and stress, deteriorated over time, from over 40% in September and October 2020 to over 50% in December 2020 and February 2021 (39). From the findings, it is suggested that teachers have been going through intensified mental health problems in the world during the pandemic largely due to growing household responsibilities (30), unpaid work overload, prolonged home confinement, sense of uncertainty over work and life, loss of loved ones during the COVID-19 pandemic as well as non-stop exposure to negative news on social and electronic media (14, 33, 38, 42). If not appropriately addressed, the academia and education system worldwide may face further challenges in continuing academic and administrative works.

Findings from the bivariate analysis showed that male teachers were experiencing more mental health problems than their female colleagues, and likewise, teachers older than 31 years of age were also suffering from depression, anxiety, and stress compared to their colleagues younger than 30 years of age. However, adjusted models of linear regression found no significant impact of sex and age on depression, anxiety, and stress. Previous studies showed more or less similar results. For example, a Chinese study noted that older teachers were more likely to experience a higher incidence of PTSD than their younger colleagues (34). However, female teachers suffered from a higher incidence of PTSD than their male colleagues. Other studies also correspond the same observations regarding the dynamics between age and sex structure with mental health problems among teachers during the COVID-19 (33, 35). Findings show that teachers, irrespective of age and sex, were experiencing mental health problems; however, male and older teachers showed higher symptoms of depression, anxiety, and stress. Because in Bangladesh, most of the households are headed by male, and they are solely responsible to provide basic necessities. The prolonged home confinement, together with countrywide lockdown, made it impossible for household heads, males in particular, to engage in regular income-generating activities or alternative livelihood opportunities to survive during the hardship (5, 23, 54). Therefore, male teachers may have experienced heightened mental health problems. In contrast, older people are more susceptible to the COVID-19 infection, and the death rate for older people was higher than that of younger people (55); hence, older people were experiencing more health problems. Thus, it is necessary to provide age and sex-specific mental health services to teachers at all levels, both in Bangladesh and other developing and developed countries. It should be noted that the prolonged COVID-19 pandemic could generate similar situations across the globe.

This study also found that teachers working at colleges and universities were less likely to suffer from depression, anxiety, and stress compared to schoolteachers. Previous studies also showed that school teachers were more likely to suffer from mental health problems during the COVID-19 than their contemporary college and university teachers (35, 56). The higher mental health issues among school teachers could be generated either from their low socioeconomic status (57, 58) to deal with the COVID-19 induced unstable socioeconomic conditions or from the additional academic burdens following the sudden switch from traditional lecture-theater based education to online platform-based education (37, 40). Considering the situation, it is recommended for policymakers to initiate job-specific mental health services that would take into account the socioeconomic condition as well as work-related burdens when implementing different measures to deal with mental health issues, especially during the outbreak of a disease like the COVID-19.

The current study also exhibited that teachers with better health status and greater usage of social and electronic media had lower chances of experiencing depression, anxiety, and stress. These results complement the existing literature that showed people with physical illness were more likely to suffer from depression, anxiety, and stress than people without any physical ailments (33, 41). Regarding social and electronic media usage, previous studies showed that over-exposure to information during the COVID-19 increased mental health problems, including depression and anxiety (13, 14). However, the insights of the teachers gained from work experience and long educational history could allow them to differentiate between “information” and “misinformation,” and it may help them to be more conscious of the risk of the COVID-19.

Besides, the current study showed a positive relationship between fear of COVID-19 and the heightened mental health problems among teachers, and such a result adheres with recent studies conducted during the COVID-19 pandemic (7). For example, a study in China observed that people living with high fear of COVID-19 showed greater symptoms of anxiety (35). Another study also found that fear of COVID-19 directly or indirectly influenced the incidence of PTSD among teachers in China (59). The physical illness or the experience of COVID-19 infection by the teachers or by their family members could trigger the fear of COVID-19 (33, 38). This study, however, did not investigate the influence of COVID-19 infection on fear of COVID-19; therefore, it suggests further research to assess how the experience of teachers shaped their mental health problems.

## Strengths and Limitations

This study has specific strong points. To our knowledge, this is the only study that explored the prevalence of depression, anxiety, and stress among teachers from all the divisions of Bangladesh during the COVID-19 pandemic. In this study, data were collected through the online platform to maintain the “social distancing” and reduce the risk of “human-to-human” infections. Besides, data regarding the teachers’ mental health were collected through a globally approved and reliable standardized questionnaire – DASS 21. Nevertheless, some limitations should be considered. This is a cross-sectional study; therefore, causality cannot be established. The participants were teachers, and it may limit the generalizability of the findings to other professional groups. The sample was selected using non-probability sampling, where the participants self-evaluated their mental health status, and such approaches may also limit the generalizability of the results. Moreover, the adjusted models identified some crucial determinants of teachers’ mental health, yet there may be a possibility of residual confounding; thus, more extensive research on a nationally representative population is required.

## Conclusion and Recommendations

The COVID-19 pandemic led to a heightened prevalence of depression, anxiety, and stress among teachers in Bangladesh. It is apparent that age, sex, residence, health condition as well as exposure to social and electronic media significantly influenced the incidence of mental health problems among teachers. Based on the outcomes of the study, it is strongly recommended that the government and its policymakers should devise effective measures to assess the mental health problems of teachers at individual, community, and institutional levels. The concerned authority should implement mental health services integrating individuals, close-relationship, and relevant other socioeconomic and politico-cultural factors to detect and minimize mental health problems during the COVID-19 pandemic and similar other emergency and non-emergency situations to ensure quality education, including the on-campus and off-campus/online education. Moreover, the authority should plan and implement programs aiming to establish and strengthen mental health services in each university, and a specific mental health center for cluster of schools and college. Because it is well evident that teachers’ mental health influences the wellbeing of their students as well as the guardians and the development of a nation.

## Data Availability Statement

The raw data supporting the conclusions of this article will be made available by the authors, without undue reservation.

## Ethics Statement

The studies involving human participants were reviewed and approved by Khulna University Ethical Clearance Committee. Written informed consent for participation was not required for this study in accordance with the national legislation and the institutional requirements.

## Author Contributions

MTH: conceptualization, investigation, data curation, formal analysis, methodology, resources, software, and writing – original draft. MAI, NJ, and MN: data curation, formal analysis, software, and writing – original draft. MS, MR, MMI, MZH, and MNI: investigation and resources. FD, KH, RA, LS, ZM, and MNI: resources and writing – original draft. All authors contributed to the article and approved the submitted version.

## Conflict of Interest

The authors declare that the research was conducted in the absence of any commercial or financial relationships that could be construed as a potential conflict of interest.

## Publisher’s Note

All claims expressed in this article are solely those of the authors and do not necessarily represent those of their affiliated organizations, or those of the publisher, the editors and the reviewers. Any product that may be evaluated in this article, or claim that may be made by its manufacturer, is not guaranteed or endorsed by the publisher.
